# Supplementary figures and images for: Occupations on the map: Using a super learner algorithm to downscale labor statistics
Source: PLoS One. 2022 Dec 7;17(12):e0278120. doi: 10.1371/journal.pone.0278120 (PMC9728836; doi:10.1371/journal.pone.0278120)

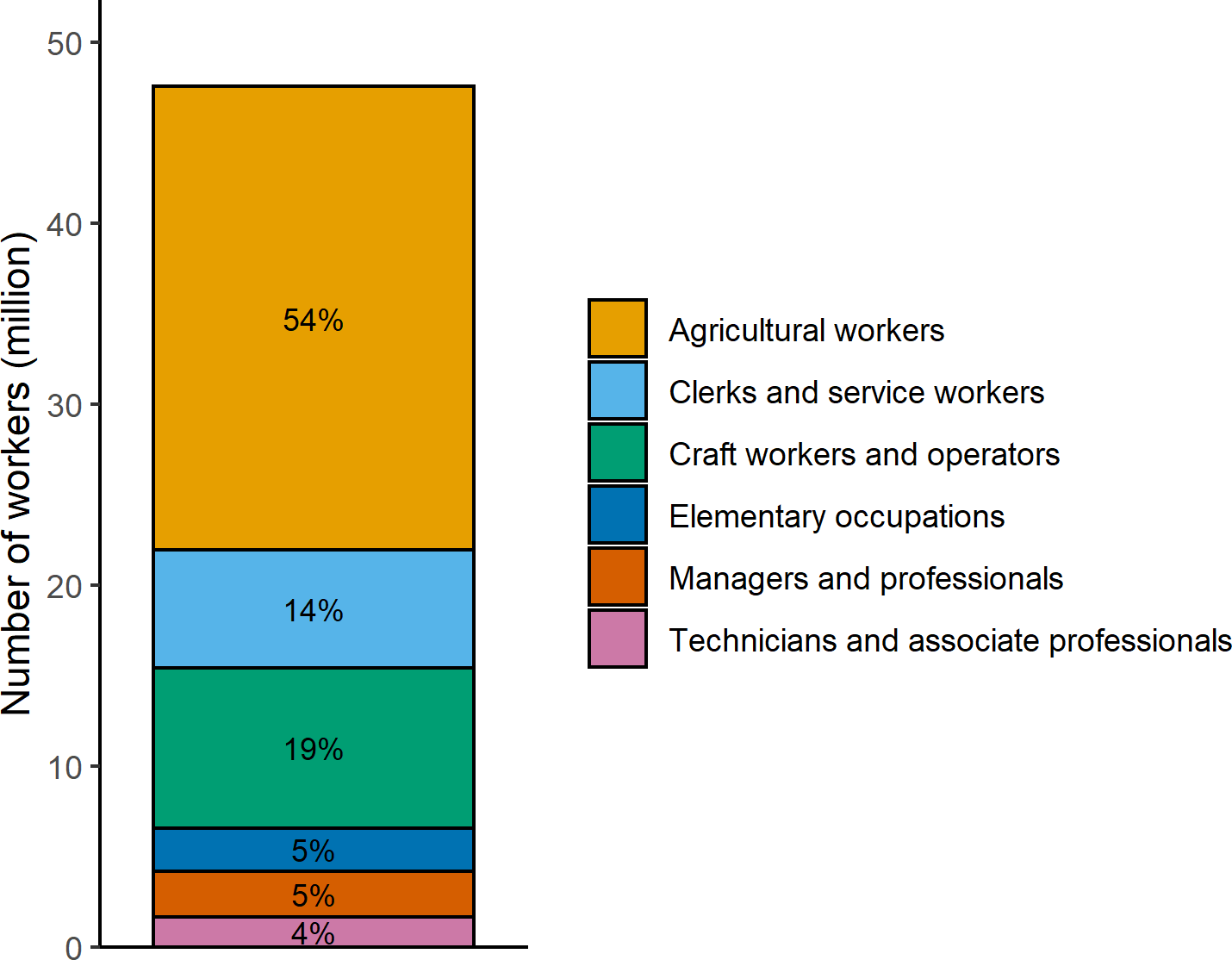

Supplement: S1 Fig — Source: Minnesota Population Center. Integrated Public Use Microdata Series, International: Version 7.2 [dataset]. Minneapolis, MN: IPUMS; 2019. https://doi.org/10.18128/D020.V7.2 for labor force participation rate and occupation shares, and Pezzulo et al. (2017) for working age population. (PNG) [file pone.0278120.s001.png]

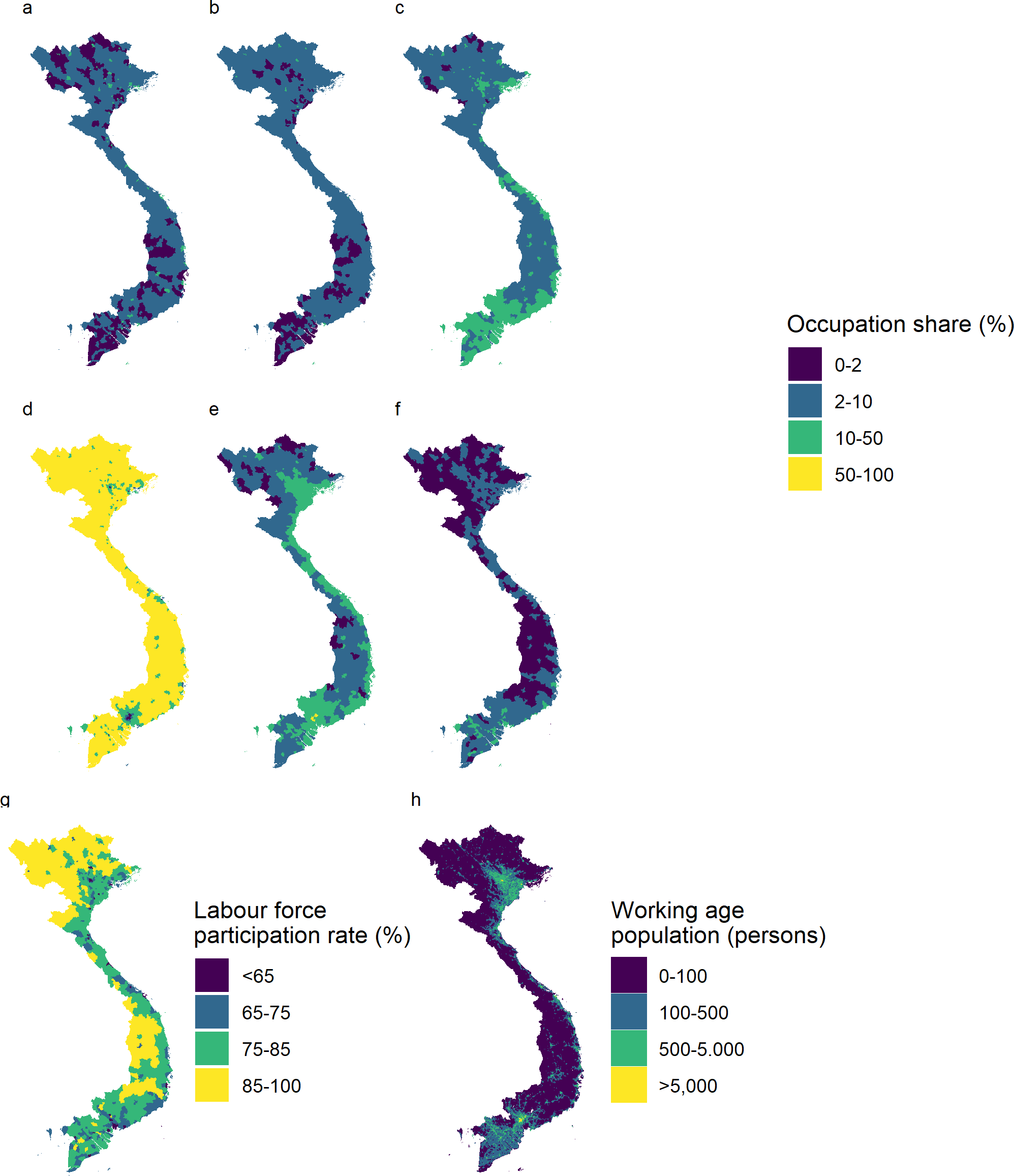

Supplement: S2 Fig — District-level information: occupation shares for (a) Managers and professionals, (b) Technicians and associate professionals, (c) Clerks and service workers, (d) Agricultural workers, (e) Craft workers and operators, (f) Elementary occupations, (g) Labor force participation rate and (h) Working age population in Vietnam for the year 2009. Source: Minnesota Population Center. Integrated Public Use Microdata Series, International: Version 7.2 [dataset]. Minneapolis, MN: IPUMS; 2019. https://doi.org/10.18128/D020.V7.2 for labor force participation rate and occupation shares, and Pezzulo et al. (2017) for working age population. (PNG) [file pone.0278120.s002.png]

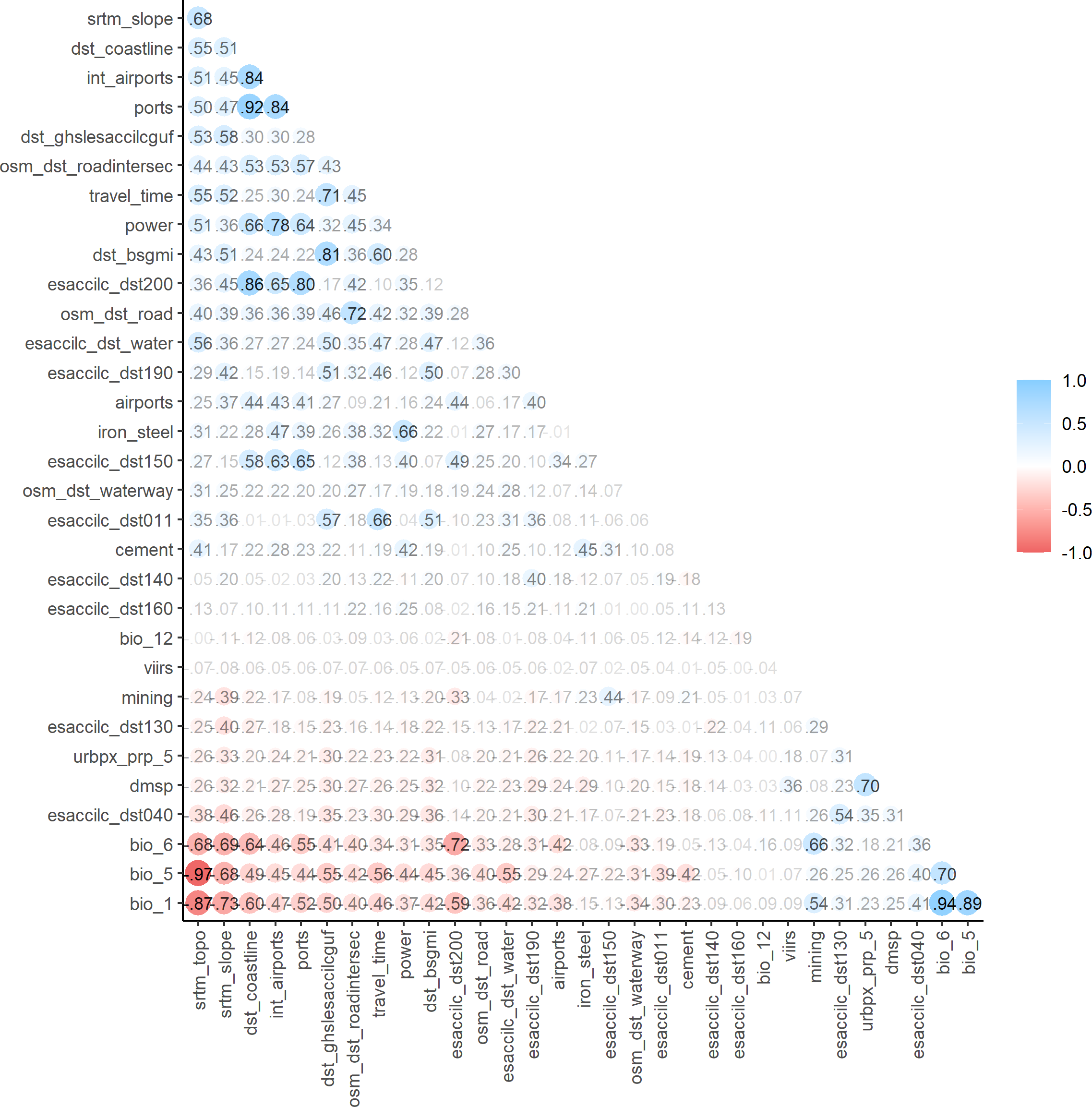

Supplement: S3 Fig — After normalization and Yeo-Johnson power transformation, we used step_corr(., threshold = .7) from the R recipes package to remove all predictors with an absolute correlation equal or larger than 0.7. Consequently, 14 predictors were removed from the analysis (bio_1, bio_5, bio_6, dmsp, dst_bsgmi, dst_ghslesaccilcguf, esaccilc_dst040, int_airports, osm_dst_road, osm_dst_roadintersec, srtm_slope, srtm_topo, travel_time, viirs), leaving 18 predictors that were used as final input. (PNG) [file pone.0278120.s003.png]

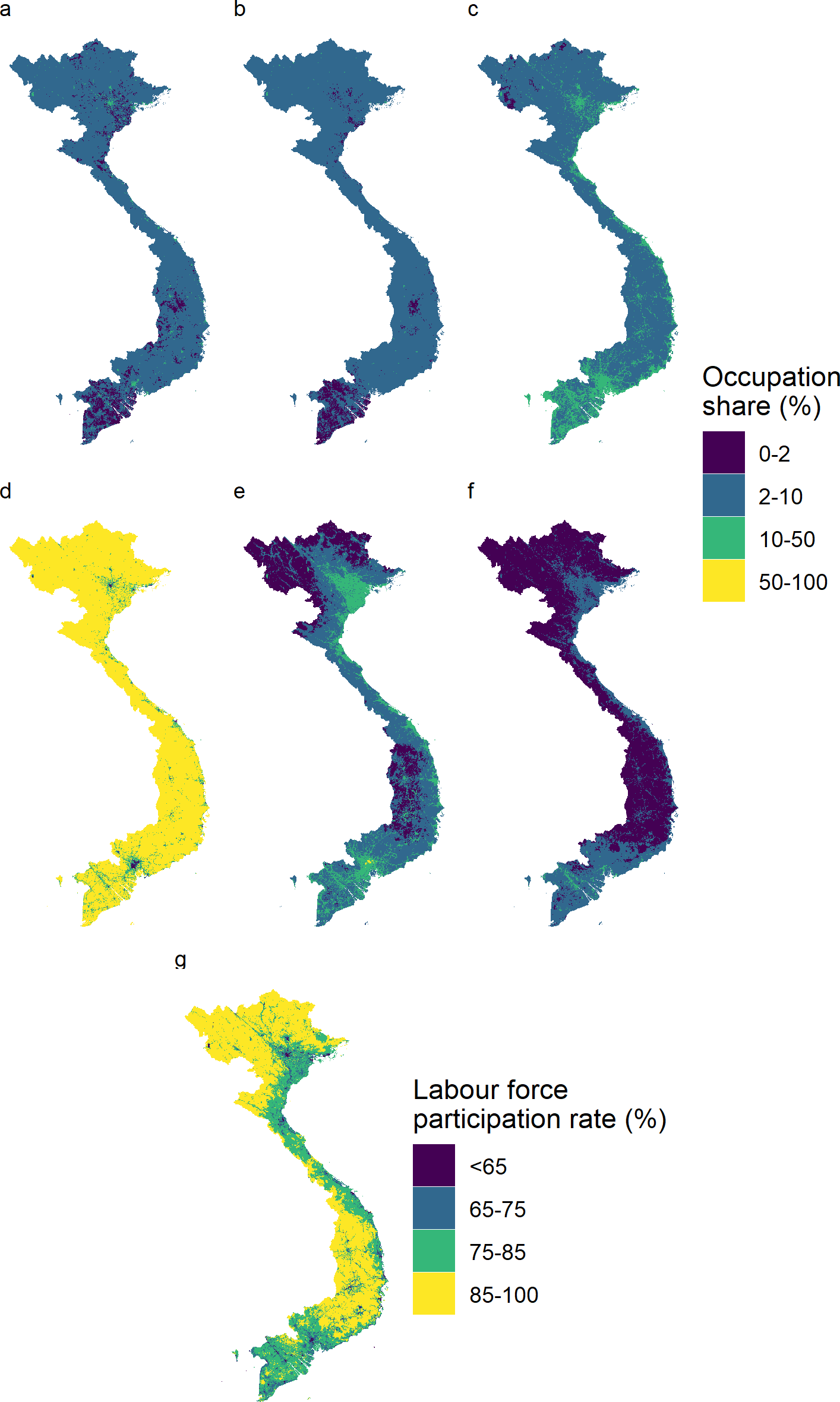

Supplement: S4 Fig — Super learner results for (a) Managers and professionals, (b) Technicians and associate professionals, (c) Clerks and service workers, (d) Agricultural workers, (e) Craft workers and operators, (f) Elementary occupations and (g) Labor force participation rate. (PNG) [file pone.0278120.s004.png]

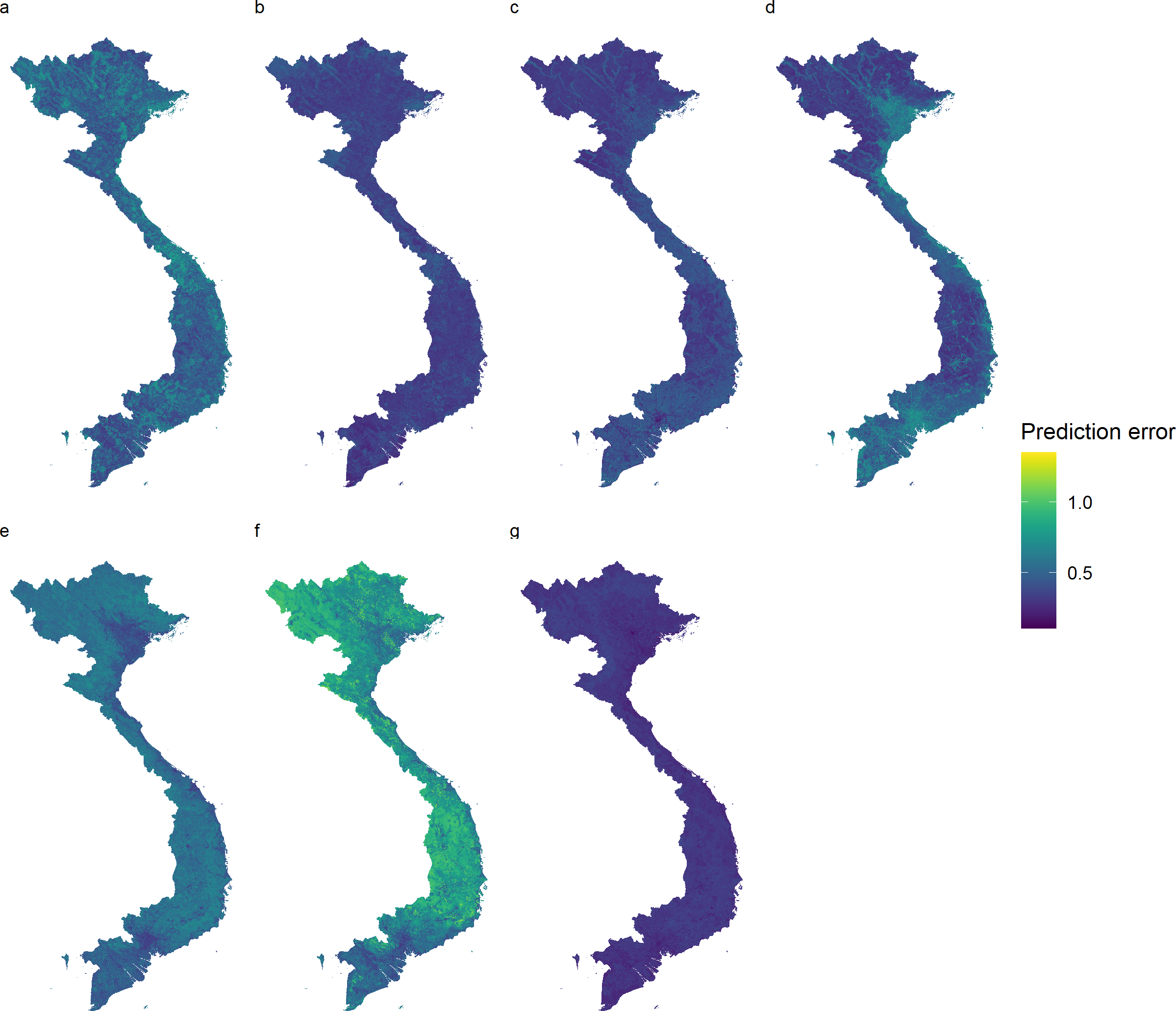

Supplement: S5 Fig — Prediction errors for (a) Managers and professionals, (b) Technicians and associate professionals, (c) Clerks and service workers, (d) Agricultural workers, (e) Craft workers and operators, (f) Elementary occupations and (g) Labor force participation rate. Prediction errors are logit transformed values, which are provided with a probability of 67%, which is the 1 standard deviation upper and lower prediction interval. (PNG) [file pone.0278120.s005.png]

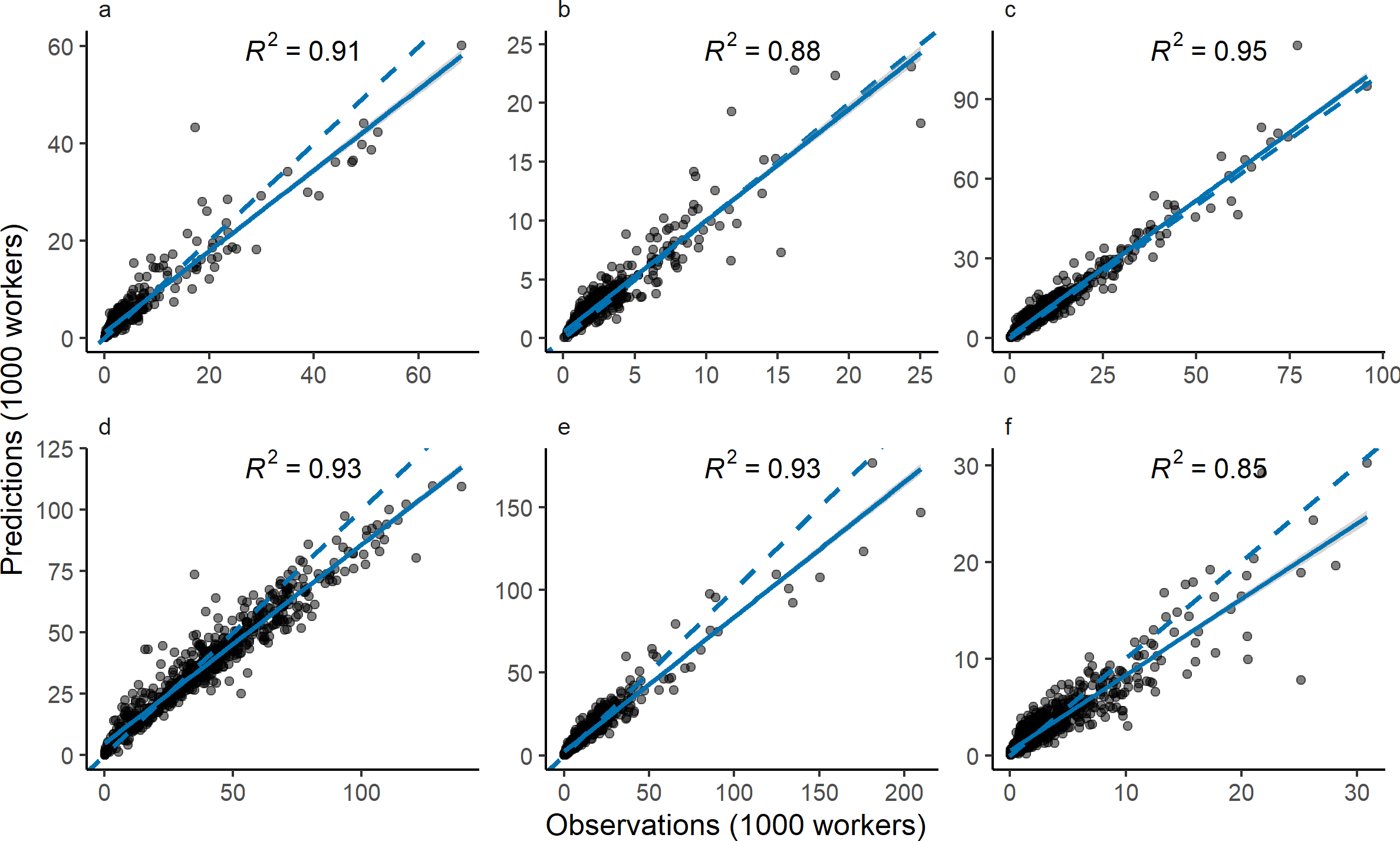

Supplement: S6 Fig — District-level comparison between observations and super learner predictions for (a) Managers and professionals, (b) Technicians and associate professionals, (c) Clerks and service workers, (d) Agricultural workers, (e) Craft workers and operators and (f) Elementary occupations. Dashed blue line represents the 1:1 line. Solid blue line indicates the regression line, with 95% confidence intervals in grey. District-level observations on the number of workers are calculated by multiplying district-level data on occupation share, labor force participation and working age population (aggregated from grid-level values), depicted in S2 Fig. (PNG) [file pone.0278120.s006.png]
