# Supplementary material for: Occupations on the map: Using a super learner algorithm to downscale labor statistics
Source: PLoS One. 2022 Dec 7;17(12):e0278120. doi: 10.1371/journal.pone.0278120 (PMC9728836; doi:10.1371/journal.pone.0278120)
Supplement: S1 Table — (PDF) [file pone.0278120.s007.pdf]

Abbreviation	Skill level	ISCO-08 Major Group	ISCO-08 description
Managers and professionals	3+4	1, 2	Legislators, senior officials and managers; professionals
Technicians and associate professionals	3	3	Technicians and associate professionals
Clerks and service workers	2	4,5	Clerical support workers; service and sales workers
Agricultural workers	2	6	Skilled agricultural, forestry and fishery workers
Craft workers and operators	2	7, 8	Craft and related trades workers; plant and machine operators, and assemblers
Elementary occupations	1	9	Elementary occupations

Table S1: Main occupation categories based on the International Classification of Occupations 08 (ILO 2012)

References

ILO. 2012. *International Standard Classification of Occupations. Volume 1: Structure, group definitions and correspondence tables.* Geneva, Switzerland: International Labour Office.
