# Supplementary material for: Occupations on the map: Using a super learner algorithm to downscale labor statistics
Source: PLoS One. 2022 Dec 7;17(12):e0278120. doi: 10.1371/journal.pone.0278120 (PMC9728836; doi:10.1371/journal.pone.0278120)
Supplement: S2 Table — (PDF) [file pone.0278120.s008.pdf]

Predictor variable	Description	Source
**Land cover**		
dst_coastline	Distance to open-water coast-line 2000-2020	Lloyd et al. (2019), worldpop.org
esaccilc_dst_water	Distance to ESA-CCI-LC inland waters 2000-2012	Lloyd et al. (2019), worldpop.org
esaccilc_dst011	Distance to ESA-CCI-LC cultivated area edges 2009	Lloyd et al. (2019), worldpop.org
esaccilc_dst040	Distance to ESA-CCI-LC woody-tree area edges 2009	Lloyd et al. (2019), worldpop.org
esaccilc_dst130	Distance to ESA-CCI-LC shrub area edges 2009	Lloyd et al. (2019), worldpop.org
esaccilc_dst140	Distance to ESA-CCI-LC herbaceous area edges 2009	Lloyd et al. (2019), worldpop.org
esaccilc_dst150	Distance to ESA-CCI-LC sparse vegetation area edges 2009	Lloyd et al. (2019), worldpop.org
esaccilc_dst160	Distance to ESA-CCI-LC aquatic vegetation area edges 2009	Lloyd et al. (2019), worldpop.org
esaccilc_dst200	Distance to ESA-CCI-LC bare area edges 2009	Lloyd et al. (2019), worldpop.org
**Night lights**		
viirs	VIIRS night-time lights 2012	Lloyd et al. (2019), worldpop.org
dmsp	DMSP-OLS night-time lights 2009	Lloyd et al. (2019), worldpop.org
**Topography**		
srtm_slope	SRTM slope 2000	Lloyd et al. (2019), worldpop.org
srtm_topo	SRTM elevation 2000	Lloyd et al. (2019), worldpop.org
**Transport**		
osm_dst_road	Distance to OSM major roads 2016	Lloyd et al. (2019), worldpop.org
osm_dst_roadintersec	Distance to OSM major roads intersections 2016	Lloyd et al. (2019), worldpop.org
osm_dst_waterway	Distance to OSM major water ways 2016	Lloyd et al. (2019), worldpop.org
int_airports	Distance to international airports 2019	World Bank (dataset/0038117/Global-Airports)
airports	Distance to airports 2017	openflights.org
ports	Distance to ports 2019	World Port Index (https://msi.nga.mil/Publications/WPI)
**Urbanization**		
esaccilc_dst190	Distance to ESA-CCI-LC artificial surface edges 2009	Lloyd et al. (2019), worldpop.org
travel_time	travel time to major cities 2015	Weiss et al. (2018)
dst_bsgmi	Built-Settlement Extents 2009	Lloyd et al. (2019), worldpop.org
dst_ghslesaccilcguf	Distance to built-settlement area edges 2012	Lloyd et al. (2019), worldpop.org
urbpx_prp_5	Proportion of built-settlement grid-cells 2012	Lloyd et al. (2019), worldpop.org
**Climate**		
bio_1	Annual mean temperature	Fick and Hijmans (2017), worldclim.org
bio_5	Max temperature of warmest month	Fick and Hijmans (2017), worldclim.org
bio_6	Min temperature of colderst month	Fick and Hijmans (2017), worldclim.org
bio_12	Annual precipitation	Fick and Hijmans (2017), worldclim.org
**Economic activity**		
power	Distance to power plants 2019	Tong et al. (2018), www.gidmodel.org.cn
iron_steel	Distance to iron and steel plants 2019	Wang et al. (2019), www.gidmodel.org.cn
cement	Distance to cement plants 2019	Liu et al. (2021), www.gidmodel.org.cn
mining	Distance to mining activities 2000-2017	Maus et al. (2020)

Table S2: List of selected predictors for the machine learning models

References

Fick, Stephen E., and Robert J. Hijmans. 2017. "WorldClim 2: new 1-km spatial resolution climate surfaces for global land areas." *International Journal of Climatology* 37 (12): 4302–15. https://doi.org/10.1002/JOC.5086.

Liu, Jun, Dan Tong, Yixuan Zheng, Jing Cheng, Xinying Qin, Qinren Shi, Liu Yan, Yu Lei, and Qiang Zhang. 2021. "Carbon and air pollutant emissions from China's cement industry 1990–2015: trends, evolution of technologies, and drivers." *Atmospheric Chemistry and Physics* 21 (3): 1627–47. https://doi.org/10.5194/acp-21-1627-2021.

Lloyd, Christopher T., Heather Chamberlain, David Kerr, Greg Yetman, Linda Pistolesi, Forrest R. Stevens, Andrea E. Gaughan, et al. 2019. "Global spatio-temporally harmonised datasets for producing high-resolution gridded population distribution datasets." *Big Earth Data* 3 (2): 108–39. https://doi.org/10.1080/20964471.2019.1625151.

Maus, Victor, Stefan Giljum, Jakob Gutschlhofer, Dieison M. da Silva, Michael Probst, Sidnei L. B. Gass, Sebastian Luckeneder, Mirko Lieber, and Ian McCallum. 2020. "A global-scale data set of mining areas." *Scientific Data* 7 (1): 289. https://doi.org/10.1038/s41597-020-00624-w.

Tong, Dan, Qiang Zhang, Steven J Davis, Fei Liu, Bo Zheng, Guannan Geng, Tao Xue, et al. 2018. "Targeted emission reductions from global super-polluting power plant units." *Nature Sustainability* 1 (1): 59–68. https://doi.org/10.1038/s41893-017-0003-y.

Wang, Xuying, Yu Lei, Li Yan, Tao Liu, Qiang Zhang, and Kebin He. 2019. "A unit-based emission inventory of SO2, NOx and PM for the Chinese iron and steel industry from 2010 to 2015." *Science of The Total Environment* 676 (August): 18–30. https://doi.org/10.1016/j.scitotenv.2019.04.241.

Weiss, D. J., A. Nelson, H. S. Gibson, W. Temperley, S. Peedell, A. Lieber, M. Hancher, et al. 2018. "A global map of travel time to cities to assess inequalities in accessibility in 2015." *Nature* 553 (7688): 333–36. https://doi.org/10.1038/nature25181.
