# Supplementary material for: Occupations on the map: Using a super learner algorithm to downscale labor statistics
Source: PLoS One. 2022 Dec 7;17(12):e0278120. doi: 10.1371/journal.pone.0278120 (PMC9728836; doi:10.1371/journal.pone.0278120)
Supplement: S1 File — (PDF) [file pone.0278120.s009.pdf]

We used the tidymodels framework (Kuhn and Johnson 2020) to implement the super learner framework. Within this framework, it is possible to select different 'engines' (i.e. R packages) for each machine learning model, each with its own set of hyperparameters that can be tuned. More specifically, we used the following engines in our model framework:

- Random forest: `ranger` (Wright and Ziegler 2017)
- Extreme gradient boosting: `xgboost` (Chen et al. 2022)
- Neural network: `nnet` (Venables and Ripley 2002)
- Polynominal and radial support vector machines: `kernlab` (Karatzoglou et al. 2004)
- Generalized linear model: `glmnet` (Friedman, Hastie, and Tibshirani 2010)

To improve transparency and model comparison (Grekousis 2019), Tables S3-S9 provide additional detail on the hyperparameters that were tuned for each of the engines. More details can be found in the package documentation.

n	member	rmse	rsq	weight	hidden_units	penalty	epochs	cost	rbf_sigma	trees	min_n	tree_depth	learn_rate	loss_reduction	sample_size
1	ml_xgboost_1_04	0.4428	0.6907	0.371						1836	6	12	0.027	0.001	0.880
2	ml_xgboost_1_25	0.4509	0.6748	0.140						1988	33	11	0.248	2.808	0.734
3	ml_xgboost_1_27	0.4538	0.6842	0.046						606	36	14	0.073	0.000	0.384
4	ml_xgboost_1_30	0.4612	0.6801	0.056						219	40	8	0.107	0.000	0.456
5	ml_neural_network_1_04	0.5271	0.5865	0.097	9	0.265	938								
6	ml_neural_network_1_26	0.5453	0.6387	0.008	6	0.000	516								
7	ml_svm_radial_1_11	0.6112	0.5122	0.015				1.825	0.869						
8	ml_neural_network_1_15	0.7295	0.4339	0.011	20	0.000	679								
9	ml_neural_network_1_08	0.7825	0.0287	0.100	1	0.000	913								
10	ml_neural_network_1_05	0.8579	0.3274	0.044	16	0.000	783								
11	ml_neural_network_1_02	0.8669	0.3735	0.070	20	0.000	874								
12	ml_neural_network_1_21	0.8971	0.3001	0.052	23	0.000	593								
13	ml_neural_network_1_07	0.9028	0.3275	0.050	25	0.000	501								

Table S3: Hyperparameters for managers and professionals super learner model members, sorted by rmse.

n	member	rmse	rsq	weight	hidden_units	penalty	epochs	cost	degree	mtry	min_n	regularization	trees	tree_depth	learn_rate	loss_reduction	sample_size
1	ml_xgboost_1_04	0.3257	0.5836	0.108							6		1836	12	0.027	0.001	0.880
2	ml_random_forest_1_06	0.3285	0.5657	0.016						27	5	0.994					
3	ml_random_forest_1_14	0.3354	0.5451	0.133						15	35	0.104					
4	ml_random_forest_1_20	0.3354	0.5429	0.009						7	39	0.064					
5	ml_random_forest_1_12	0.3380	0.5413	0.001						13	13	0.803					
6	ml_random_forest_1_25	0.3394	0.5332	0.001						10	24	0.096					
7	ml_xgboost_1_16	0.3421	0.5613	0.310							22		912	4	0.141	0.000	0.568
8	ml_xgboost_1_27	0.3436	0.5378	0.214							36		606	14	0.073	0.000	0.384
9	ml_neural_network_1_03	0.3553	0.5260	0.009	3	0.002	430										
10	ml_svm_poly_1_12	0.4190	0.5020	0.055				0.408	2								
11	ml_svm_poly_1_04	0.4294	0.4913	0.001				0.792	2								
12	ml_neural_network_1_11	0.4551	0.3936	0.084	10	0.019	217										
13	ml_neural_network_1_02	0.6626	0.2250	0.063	20	0.000	874										
14	ml_neural_network_1_28	0.7616	0.2521	0.022	15	0.008	606										

Table S4: Hyperparameters for technicians and associate professionals super learner model members, sorted by rmse.

n	member	rmse	rsq	weight	hidden_units	penalty	epochs	cost	degree	trees	min_n	tree_depth	learn_rate	loss_reduction	sample_size
1	ml_xgboost_1_04	0.4033	0.7755	0.241						1836	6	12	0.027	0.001	0.880
2	ml_xgboost_1_12	0.4077	0.7699	0.048						111	16	15	0.037	0.085	0.952
3	ml_xgboost_1_19	0.4111	0.7685	0.354						1703	25	11	0.017	0.000	0.858
4	ml_svm_poly_1_29	0.4784	0.6919	0.021				29.168	2						
5	ml_svm_poly_1_02	0.4792	0.6943	0.071				11.251	2						
6	ml_neural_network_1_11	0.5019	0.6931	0.070	10	0.019	217								
7	ml_neural_network_1_25	0.5022	0.6764	0.048	9	0.000	105								
8	ml_neural_network_1_13	0.5272	0.6596	0.022	17	0.030	325								
9	ml_xgboost_1_17	0.5319	0.6169	0.029						1207	23	2	0.165	0.000	0.117
10	ml_neural_network_1_28	0.5877	0.6102	0.040	15	0.008	606								
11	ml_svm_poly_1_09	0.6161	0.5615	0.016				0.034	3						
12	ml_neural_network_1_17	0.6600	0.5152	0.044	14	0.000	842								

Table S5: Hyperparameters for clerks and service workers super learner model members, sorted by rmse.

n	member	rmse	rsq	weight	hidden_units	penalty	epochs	cost	rbf_sigma	degree	mtry	min_n	regularization	trees	tree_depth	learn_rate	loss_reduction	sample_size
1	ml_xgboost_1_04	0.5279	0.9302	0.227								6		1836	12	0.027	0.001	0.880
2	ml_xgboost_1_12	0.5547	0.9278	0.113								16		111	15	0.037	0.085	0.952
3	ml_random_forest_1_12	0.5551	0.9273	0.130							13	13	0.803					
4	ml_random_forest_1_07	0.5622	0.9232	0.000							31	18	0.496					
5	ml_random_forest_1_24	0.5678	0.9235	0.025							14	11	0.299					
6	ml_neural_network_1_29	0.5929	0.9119	0.006	8	0.154	364											
7	ml_neural_network_1_14	0.5963	0.9122	0.026	13	0.063	113											
8	ml_xgboost_1_30	0.6344	0.9000	0.035								40		219	8	0.107	0.000	0.456
9	ml_xgboost_1_28	0.6757	0.8884	0.030								37		1630	12	0.022	0.000	0.245
10	ml_neural_network_1_13	0.8174	0.8353	0.000	17	0.030	325											
11	ml_xgboost_1_17	0.8219	0.8293	0.024								23		1207	2	0.165	0.000	0.117
12	ml_neural_network_1_24	0.8263	0.8345	0.023	12	0.000	306											
13	ml_svm_poly_1_29	0.9030	0.8275	0.056				29.168		2								
14	ml_svm_poly_1_09	0.9110	0.8144	0.013				0.034		3								
15	ml_neural_network_1_21	0.9616	0.7867	0.018	23	0.000	593											
16	ml_neural_network_1_17	0.9723	0.8003	0.057	14	0.000	842											
17	ml_neural_network_1_02	1.0959	0.7348	0.033	20	0.000	874											
18	ml_neural_network_1_16	1.1158	0.7570	0.126	26	0.000	760											
19	ml_neural_network_1_09	1.1439	0.7326	0.058	25	0.000	376											
20	ml_svm_radial_1_11	1.3160	0.6935	0.061				1.825	0.869									

Table S6: Hyperparameters for agricultural workers super learner model members, sorted by rmse.

n	member	rmse	rsq	weight	hidden_units	penalty	epochs	cost	rbf_sigma	mtry	min_n	regularization	trees	tree_depth	learn_rate	loss_reduction	sample_size
1	ml_xgboost_1_01	0.3974	0.8670	0.509							3		749	14	0.058	0.000	0.510
2	ml_xgboost_1_21	0.4212	0.8540	0.089							27		1515	13	0.009	0.001	0.416
3	ml_xgboost_1_16	0.4356	0.8412	0.005							22		912	4	0.141	0.000	0.568
4	ml_xgboost_1_27	0.4435	0.8389	0.059							36		606	14	0.073	0.000	0.384
5	ml_neural_network_1_11	0.4633	0.8321	0.008	10	0.019	217										
6	ml_neural_network_1_30	0.4940	0.8026	0.044	2	0.000	145										
7	ml_random_forest_1_13	0.5383	0.7580	0.008						21	34	0.318					
8	ml_random_forest_1_28	0.5383	0.7583	0.026						18	32	0.602					
9	ml_neural_network_1_26	0.5539	0.7614	0.033	6	0.000	516										
10	ml_neural_network_1_12	0.6973	0.6690	0.009	11	0.000	805										
11	ml_neural_network_1_17	0.7153	0.6489	0.038	14	0.000	842										
12	ml_neural_network_1_05	0.7277	0.6488	0.111	16	0.000	783										
13	ml_neural_network_1_28	0.8048	0.5826	0.037	15	0.008	606										
14	ml_neural_network_1_09	0.8552	0.5817	0.011	25	0.000	376										
15	ml_neural_network_1_27	0.9649	0.5112	0.019	19	0.000	664										
16	ml_svm_radial_1_27	1.0907	0.7462	0.000				0.160	0								
17	ml_svm_radial_1_26	1.0907	0.7462	0.249				0.001	0								

Table S7: Hyperparameters for craft workers and operators super learner model members, sorted by rmse.

n	member	rmse	rsq	weight	hidden_units	penalty	epochs	cost	rbf_sigma	mtry	min_n	regularization	trees	tree_depth	learn_rate	loss_reduction	sample_size
1	ml_xgboost_1_02	0.6153	0.6135	0.099							4		1446	6	0.043	0	0.587
2	ml_xgboost_1_01	0.6243	0.6009	0.263							3		749	14	0.058	0	0.510
3	ml_random_forest_1_18	0.6312	0.5904	0.144						4	7	0.444					
4	ml_svm_radial_1_23	0.6409	0.5776	0.020				5.167	0.134								
5	ml_random_forest_1_12	0.6636	0.5517	0.118						13	13	0.803					
6	ml_neural_network_1_04	0.7070	0.5323	0.180	9	0.265	938										
7	ml_neural_network_1_26	0.7423	0.5302	0.021	6	0.000	516										
8	ml_neural_network_1_05	1.2029	0.3010	0.040	16	0.000	783										
9	ml_neural_network_1_16	1.2847	0.1667	0.020	26	0.000	760										
10	ml_neural_network_1_22	1.3610	0.2343	0.008	21	0.000	571										

Table S8: Hyperparameters for elementary occupations super learner model members, sorted by rmse.

n	member	rmse	rsq	weight	hidden_units	penalty	epochs	cost	degree	mtry	min_n	regularization	trees	tree_depth	learn_rate	loss_reduction	sample_size
1	ml_xgboost_1_29	0.2653	0.7346	0.122							39		567	5	0.013	0.366	0.981
2	ml_random_forest_1_06	0.2707	0.7238	0.102						27	5	0.994					
3	ml_random_forest_1_24	0.2792	0.7045	0.049						14	11	0.299					
4	ml_xgboost_1_16	0.2825	0.7074	0.003							22		912	4	0.141	0.000	0.568
5	ml_xgboost_1_04	0.2841	0.6949	0.168							6		1836	12	0.027	0.001	0.880
6	ml_xgboost_1_01	0.2845	0.6950	0.179							3		749	14	0.058	0.000	0.510
7	ml_svm_poly_1_27	0.3211	0.6248	0.074				0.160	2								
8	ml_svm_poly_1_12	0.3227	0.6241	0.054				0.408	2								
9	ml_neural_network_1_11	0.3299	0.6203	0.048	10	0.019	217										
10	ml_svm_poly_1_29	0.3764	0.5370	0.013				29.168	2								
11	ml_neural_network_1_24	0.4763	0.4301	0.001	12	0.000	306										
12	ml_svm_poly_1_09	0.4942	0.3606	0.069				0.034	3								
13	ml_neural_network_1_19	0.5097	0.2862	0.031	15	0.000	271										
14	ml_neural_network_1_22	0.5205	0.2912	0.058	21	0.000	571										
15	ml_svm_poly_1_25	0.5573	0.2831	0.025				0.315	3								
16	ml_xgboost_1_03	0.6626	0.6940	0.033							5		316	13	0.002	0.014	0.829

Table S9: Hyperparameters for labour force participation rate super learner model members, sorted by rmse.

References

Chen, T., T. He, M. Benesty, V. Khotilovich, Y. Tang, H. Cho, K. Chen, et al. 2022. "xgboost: Extreme Gradient Boosting." https://cran.r-project.org/package=xgboost.

Friedman, Jerome, Trevor Hastie, and Robert Tibshirani. 2010. "Regularization Paths for Generalized Linear Models via Coordinate Descent." *Journal of Statistical Software* 33 (1). https://doi.org/10.18637/jss.v033.i01.

Grekousis, George. 2019. "Artificial neural networks and deep learning in urban geography: A systematic review and meta-analysis." *Computers, Environment and Urban Systems* 74 (March): 244–56. https://doi.org/10.1016/J.COMPENVURBSYS.2018.10.008.

Karatzoglou, Alexandros, Alex Smola, Kurt Hornik, and Achim Zeileis. 2004. "kernlab - An S4 Package for Kernel Methods in R." *Journal of Statistical Software* 11 (9). https://doi.org/10.18637/jss.v011.i09.

Kuhn, Max, and K. Johnson. 2020. *Feature Engineering and Selection: A Practical Approach for Predictive Models*. CRC Press.

Venables, W. N., and S. Ripley. 2002. *Modern Applied Statistics with S*. Fourth. New York: Springer.

Wright, Marvin N., and Andreas Ziegler. 2017. "ranger : A Fast Implementation of Random Forests for High Dimensional Data in C++ and R." *Journal of Statistical Software* 77 (1). https://doi.org/10.18637/jss.v077.i01.
