# Supplementary material for: Occupations on the map: Using a super learner algorithm to downscale labor statistics
Source: PLoS One. 2022 Dec 7;17(12):e0278120. doi: 10.1371/journal.pone.0278120 (PMC9728836; doi:10.1371/journal.pone.0278120)
Supplement: S2 File — Results are presented for the five super learner model members with the highest weight and for the top 10 predictors. Starting values for the horizontal bars indicate the RMSE for the full model. Predictors with the largest bars are the most important because permuting them results in higher RMSE. Error bars indicate results for 10 different permutations. (PDF) [file pone.0278120.s010.pdf]

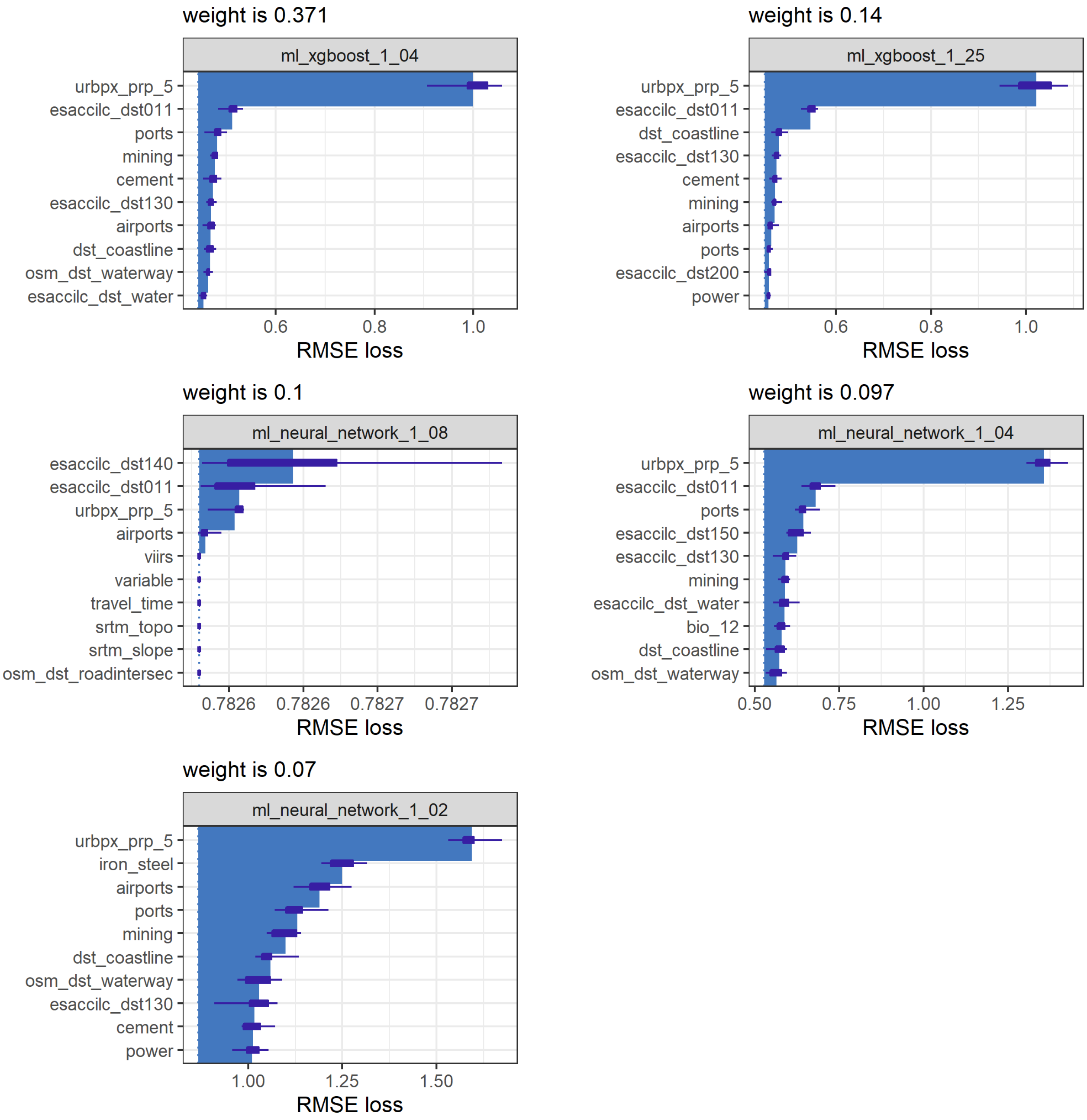

Fig. S7: Variable importance plots for managers and professionals. Results are presented for the five super learner model members with the highest weight and for the top 10 predictors. Starting values for the horizontal bars indicate the RMSE for the full model. Predictors with the largest bars are the most important because permuting them results in higher RMSE. Error bars indicate results for 10 different permutations.

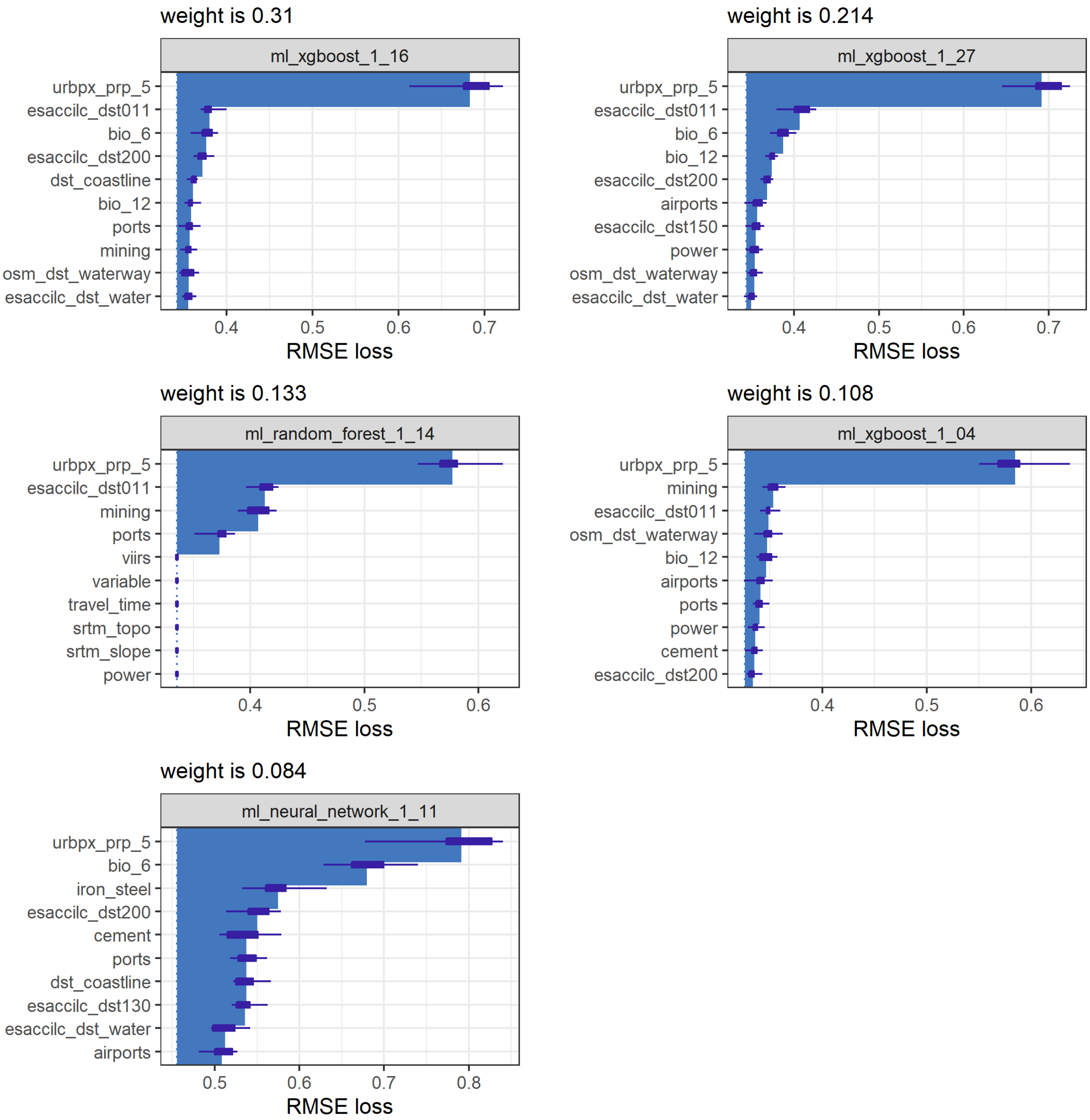

Fig. S8: Variable importance plots for technicians and associate professionals. Results are presented for the five super learner model members with the highest weight and for the top 10 predictors. Starting values for the horizontal bars indicate the RMSE for the full model. Predictors with the largest bars are the most important because permuting them results in higher RMSE. Error bars indicate results for 10 different permutations.

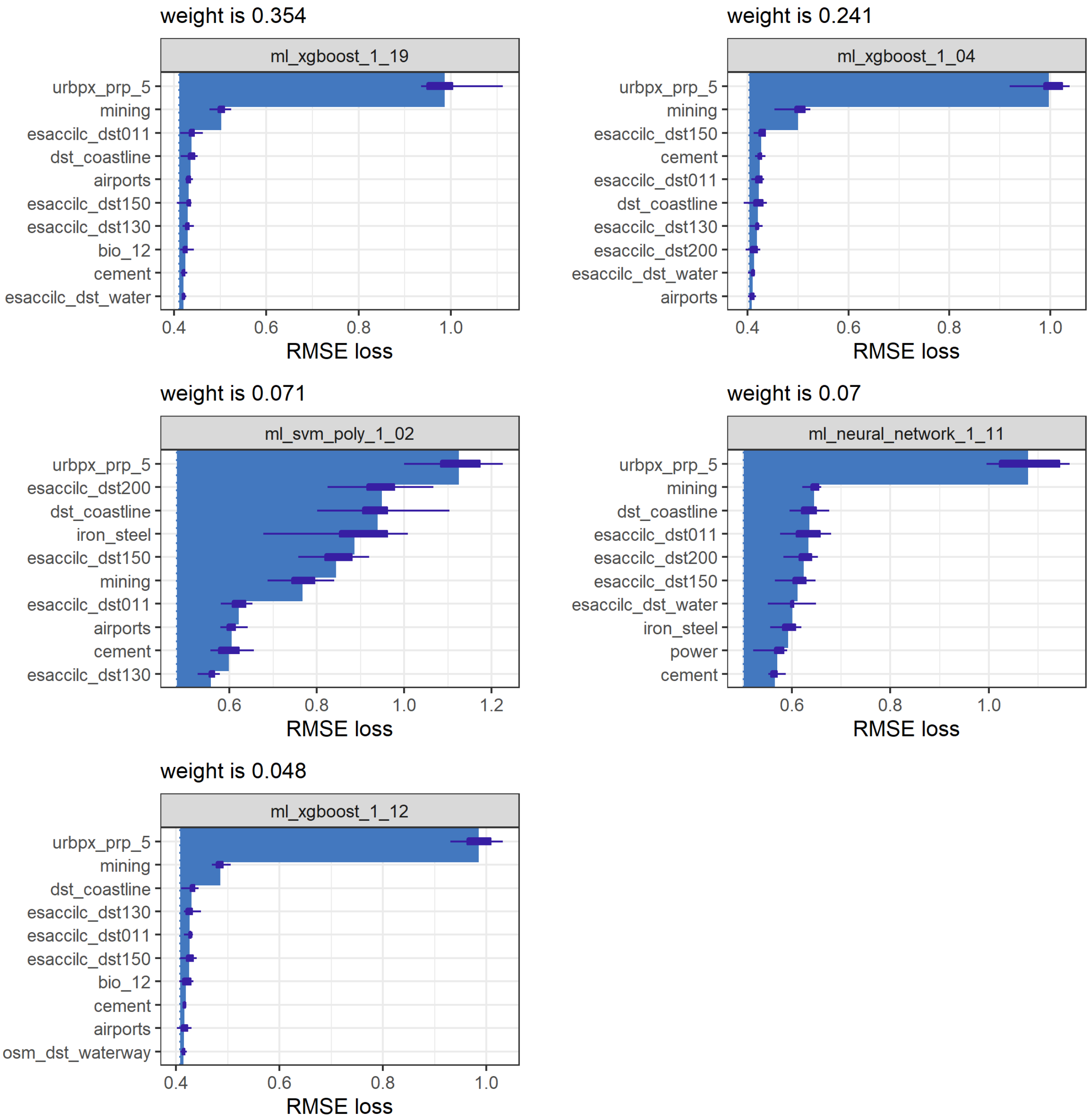

Fig. S9: Variable importance plots for clerks and service workers. Results are presented for the five super learner model members with the highest weight and for the top 10 predictors. Starting values for the horizontal bars indicate the RMSE for the full model. Predictors with the largest bars are the most important because permuting them results in higher RMSE. Error bars indicate results for 10 different permutations.

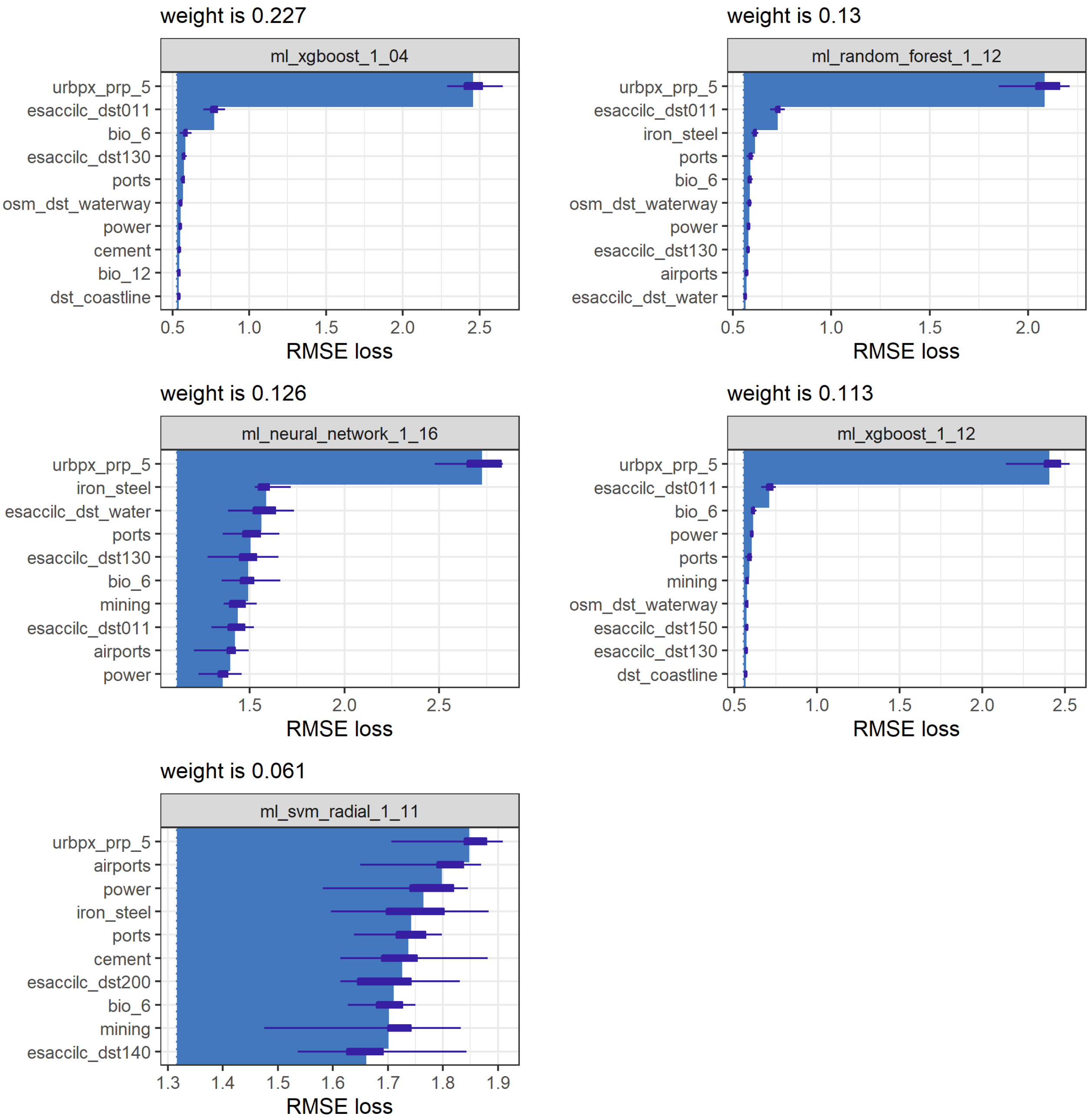

Fig. S10: Variable importance plots for agricultural workers. Results are presented for the five super learner model members with the highest weight and for the top 10 predictors. Starting values for the horizontal bars indicate the RMSE for the full model. Predictors with the largest bars are the most important because permuting them results in higher RMSE. Error bars indicate results for 10 different permutations.

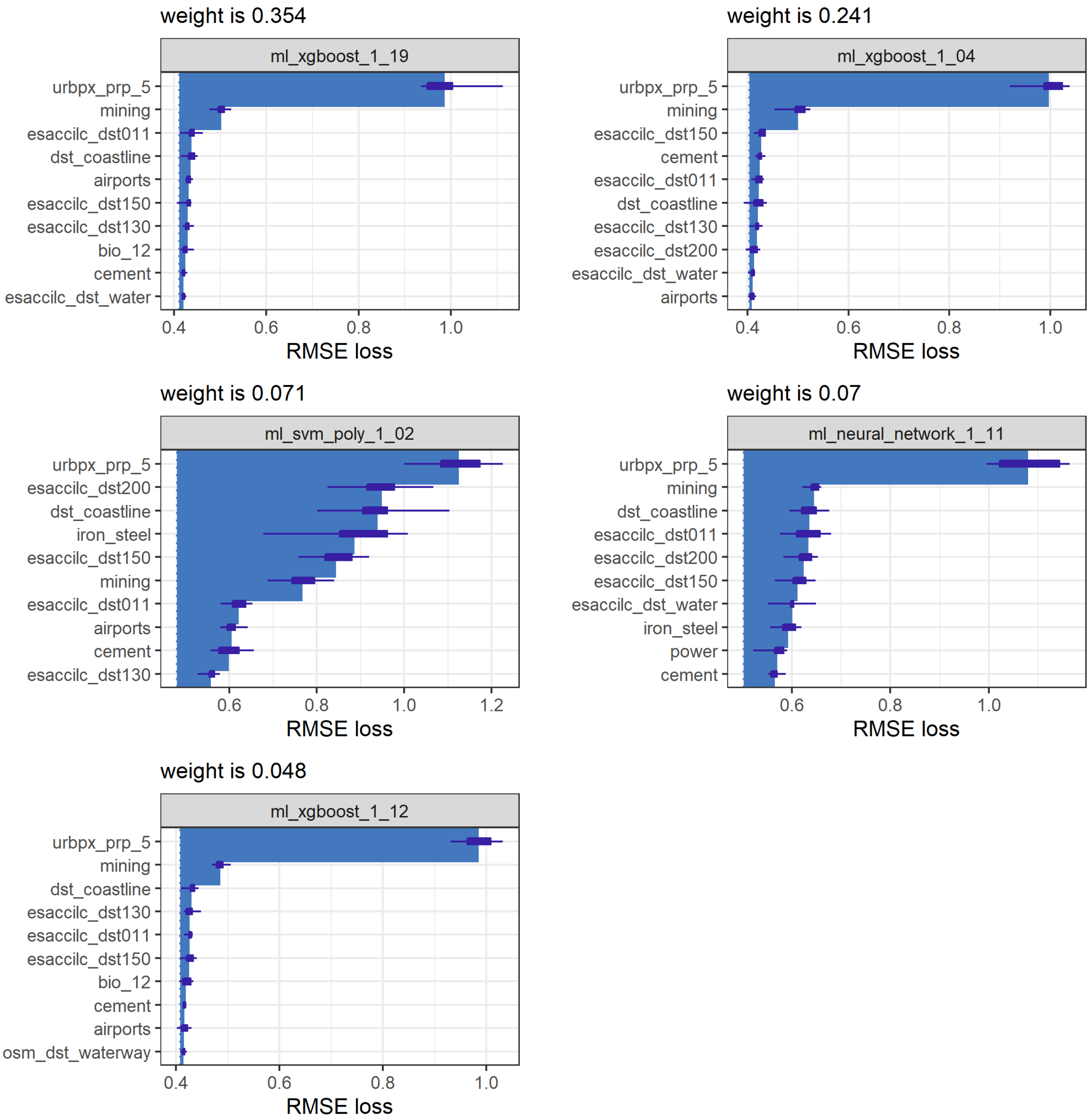

Fig. S11: Variable importance plots for craft workers. Results are presented for the five super learner model members with the highest weight and for the top 10 predictors. Starting values for the horizontal bars indicate the RMSE for the full model. Predictors with the largest bars are the most important because permuting them results in higher RMSE. Error bars indicate results for 10 different permutations.

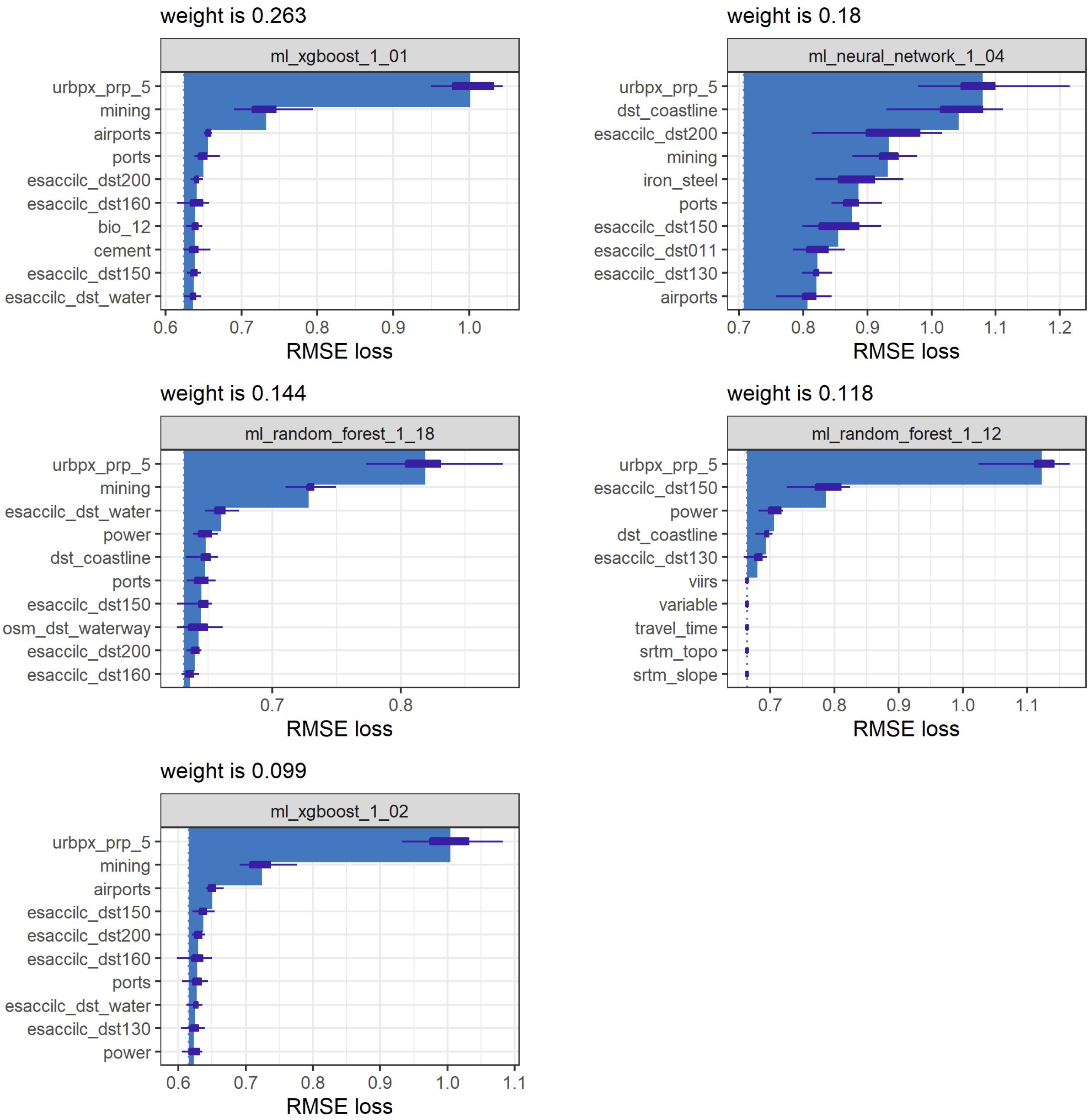

Fig. S12: Variable importance plots for elementary occupations. Results are presented for the five super learner model members with the highest weight and for the top 10 predictors. Starting values for the horizontal bars indicate the RMSE for the full model. Predictors with the largest bars are the most important because permuting them results in higher RMSE. Error bars indicate results for 10 different permutations.

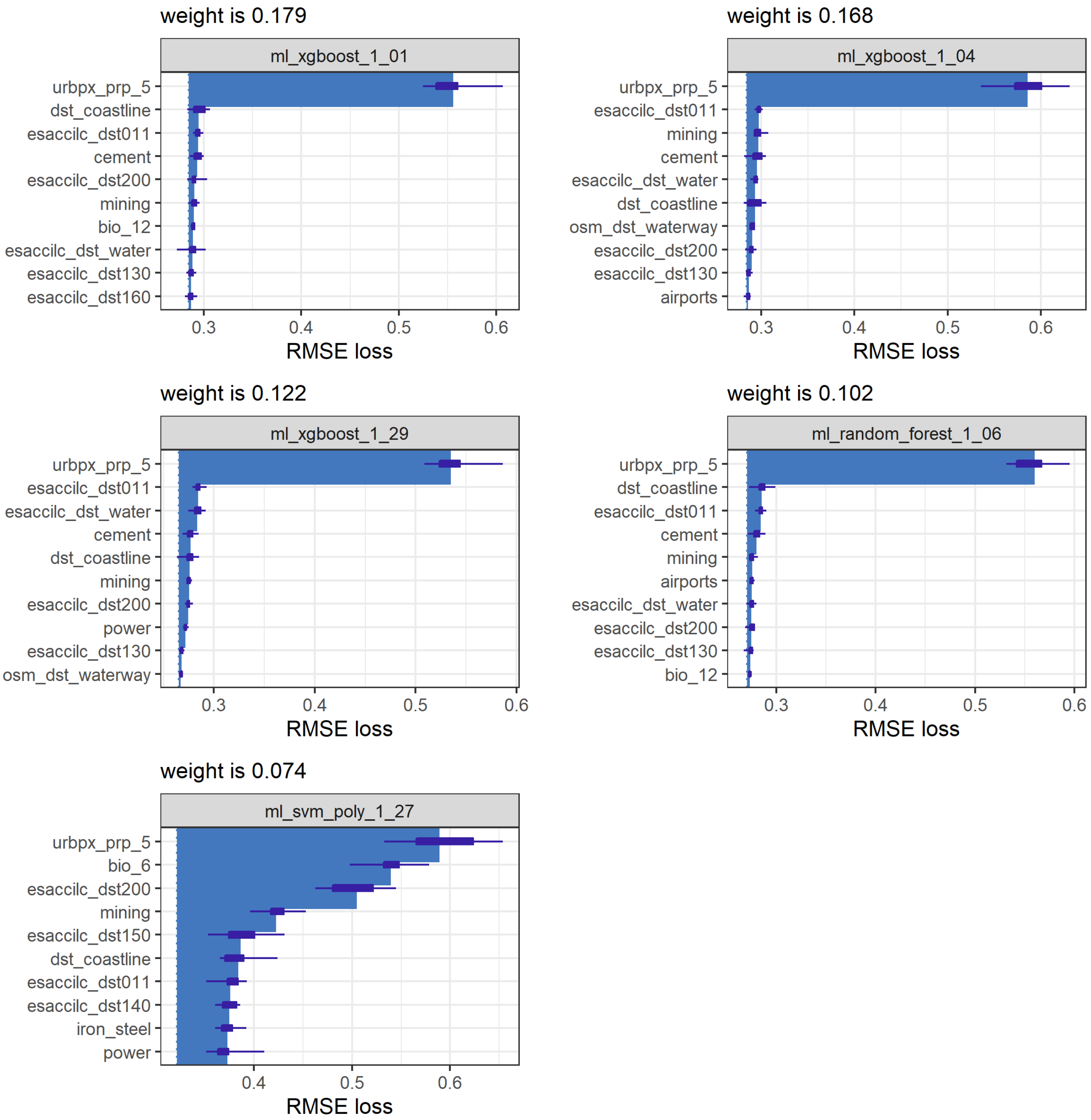

Fig. S13: Variable importance plots for labour force participation rate. Results are presented for the five super learner model members with the highest weight and for the top 10 predictors. Starting values for the horizontal bars indicate the RMSE for the full model. Predictors with the largest bars are the most important because permuting them results in higher RMSE. Error bars indicate results for 10 different permutations.
