# Supplementary material for: Occupations on the map: Using a super learner algorithm to downscale labor statistics
Source: PLoS One. 2022 Dec 7;17(12):e0278120. doi: 10.1371/journal.pone.0278120 (PMC9728836; doi:10.1371/journal.pone.0278120)
Supplement: S3 File — Results are presented for the five super learner model members with the highest weight. (PDF) [file pone.0278120.s011.pdf]

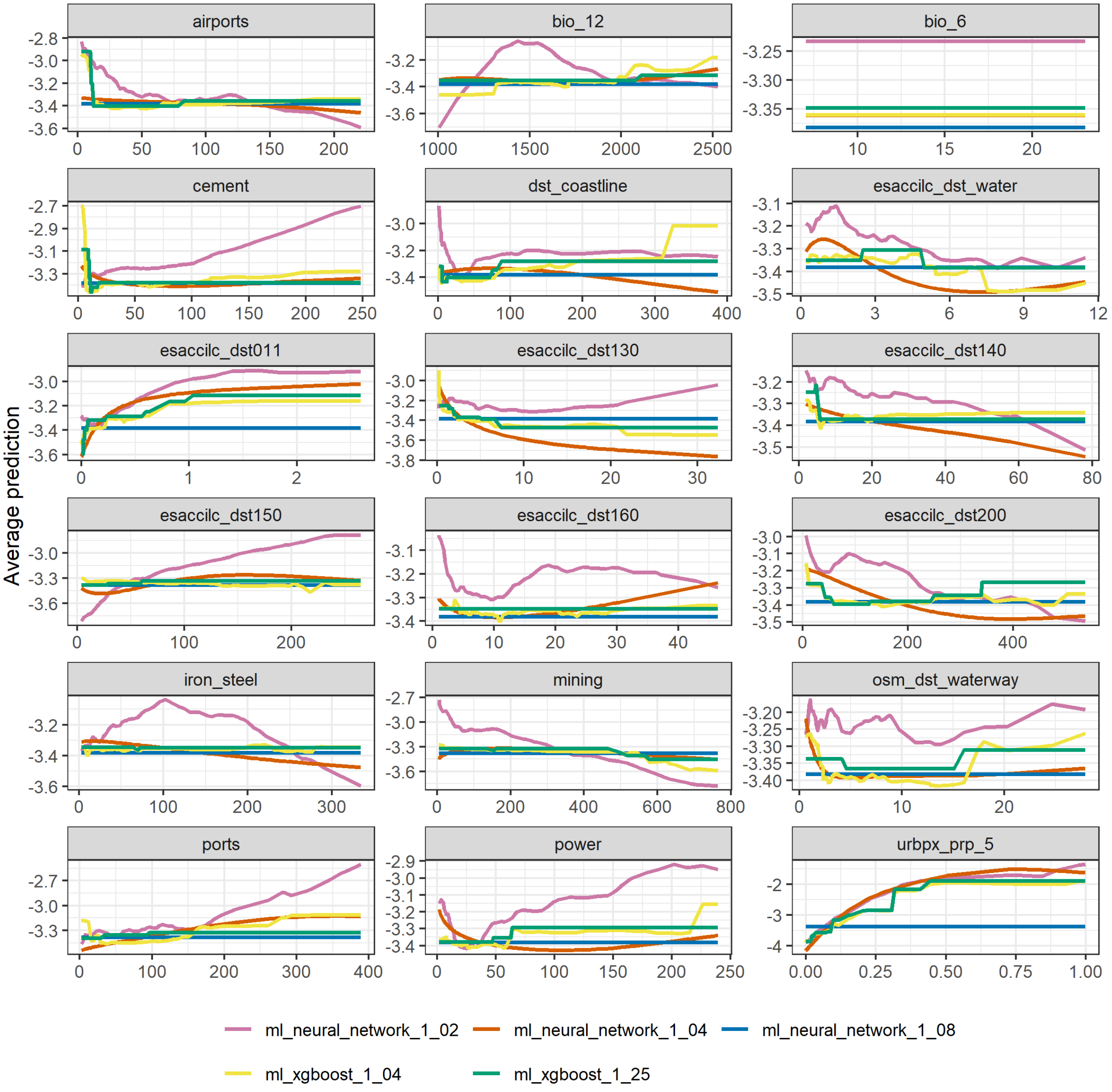

Fig. S14: Accumulated local effects plots for managers and professionals. Results are presented for the five super learner model members with the highest weight.

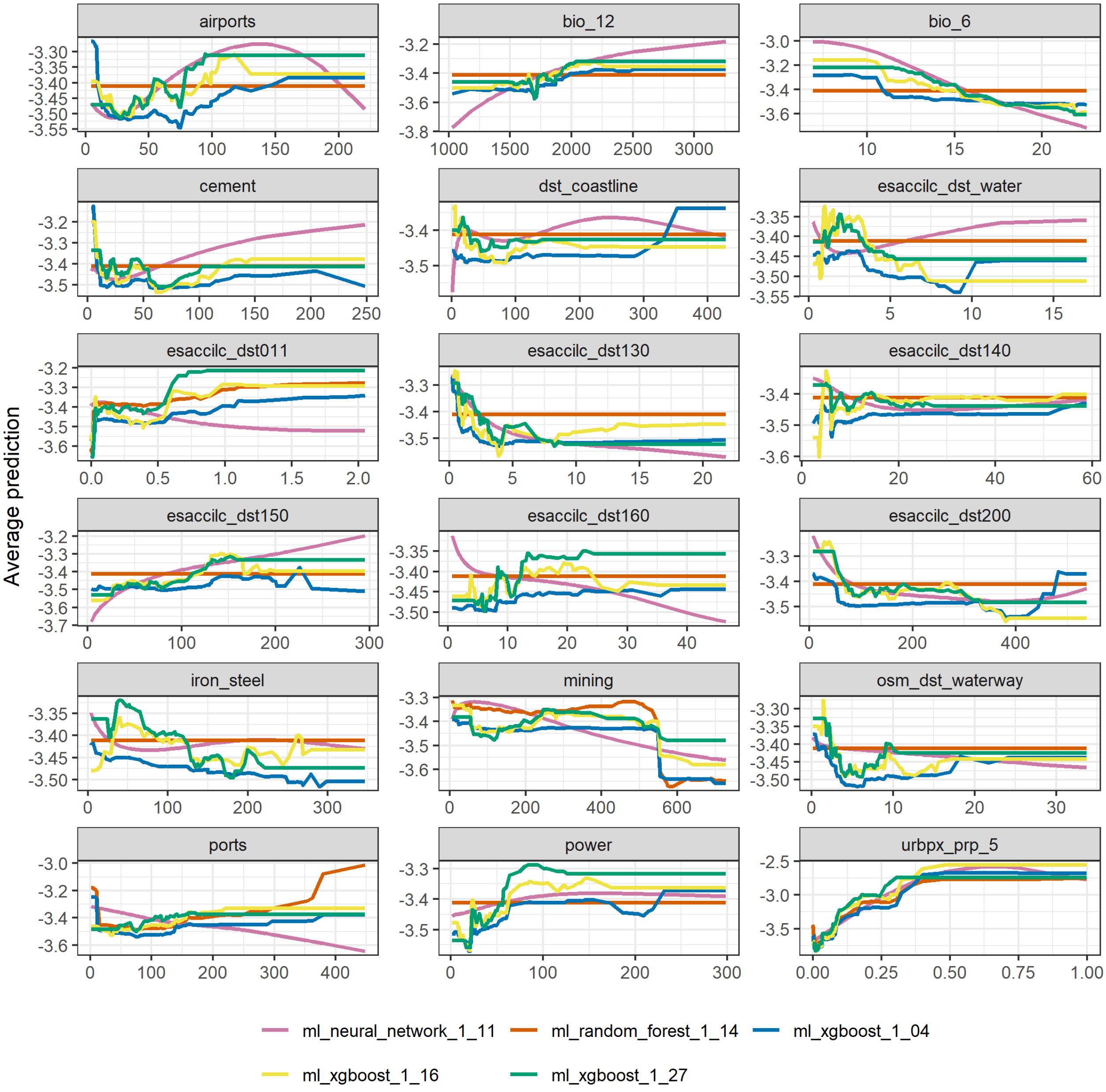

Fig. S15: Accumulated local effects plots for technicians and associate professionals. Results are presented for the five super learner model members with the highest weight.

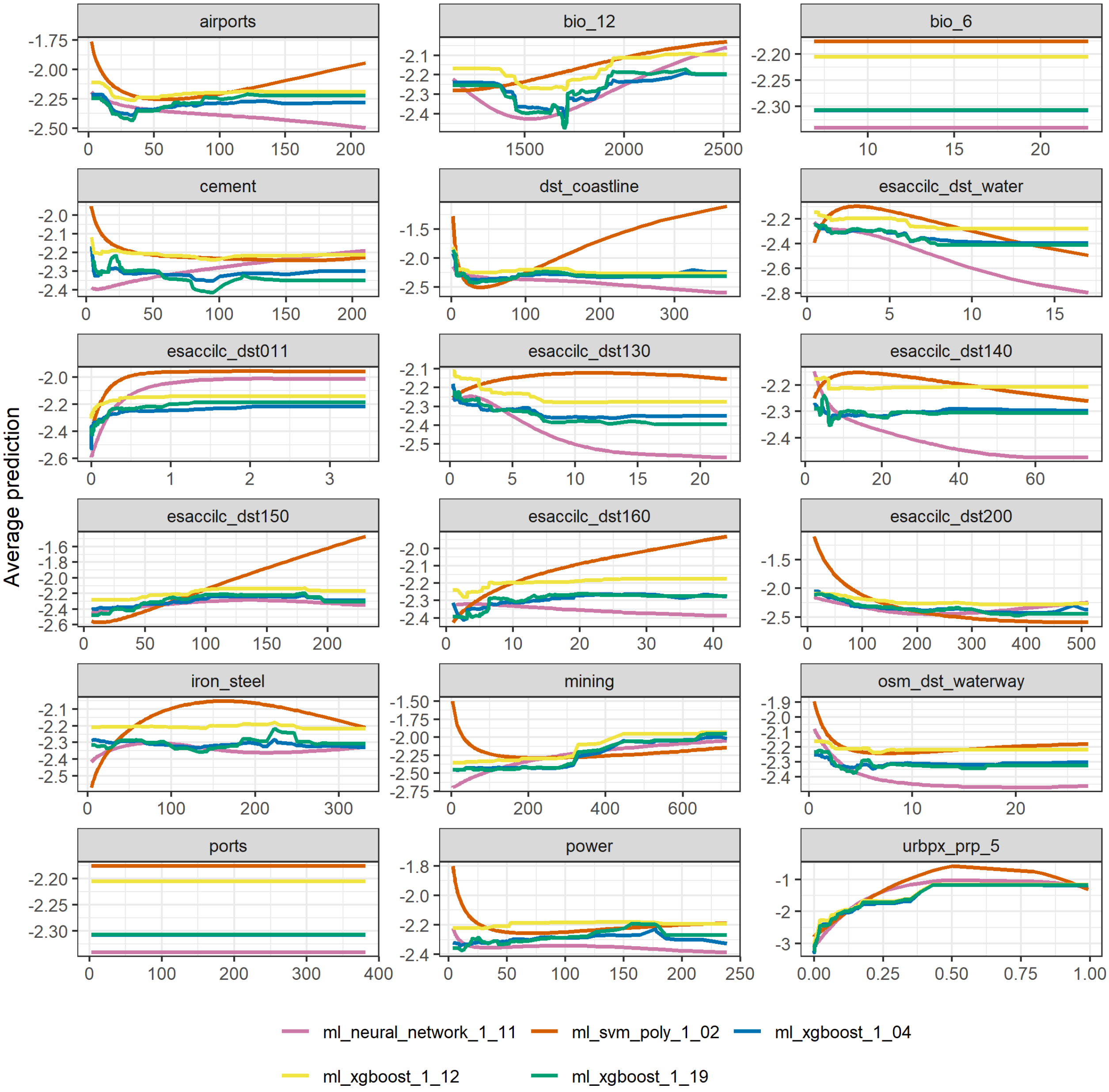

Fig. S16: Accumulated local effects plots for clerks and service workers. Results are presented for the five super learner model members with the highest weight.

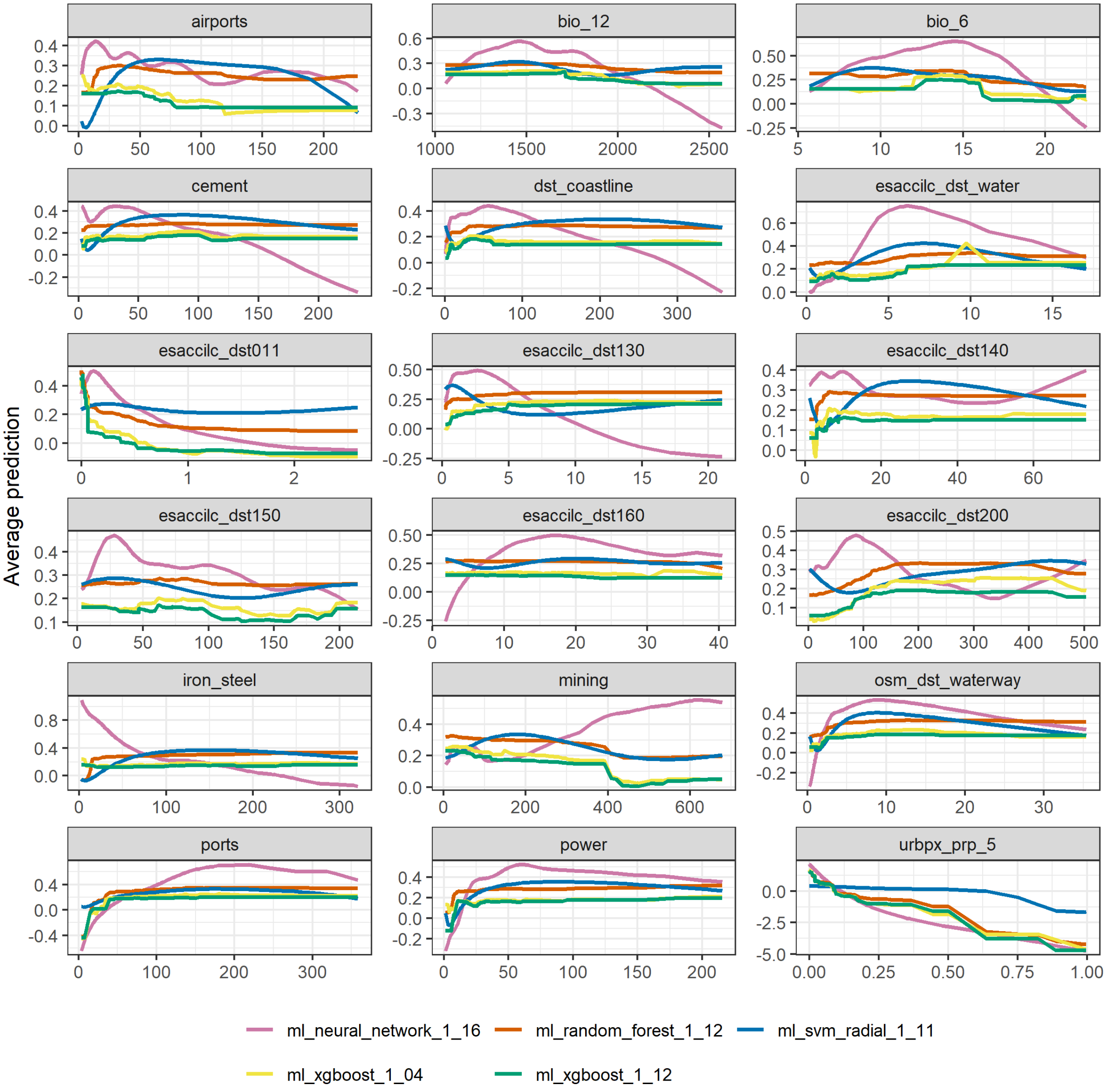

Fig. S17: Accumulated local effects plots for agricultural workers. Results are presented for the five super learner model members with the highest weight.

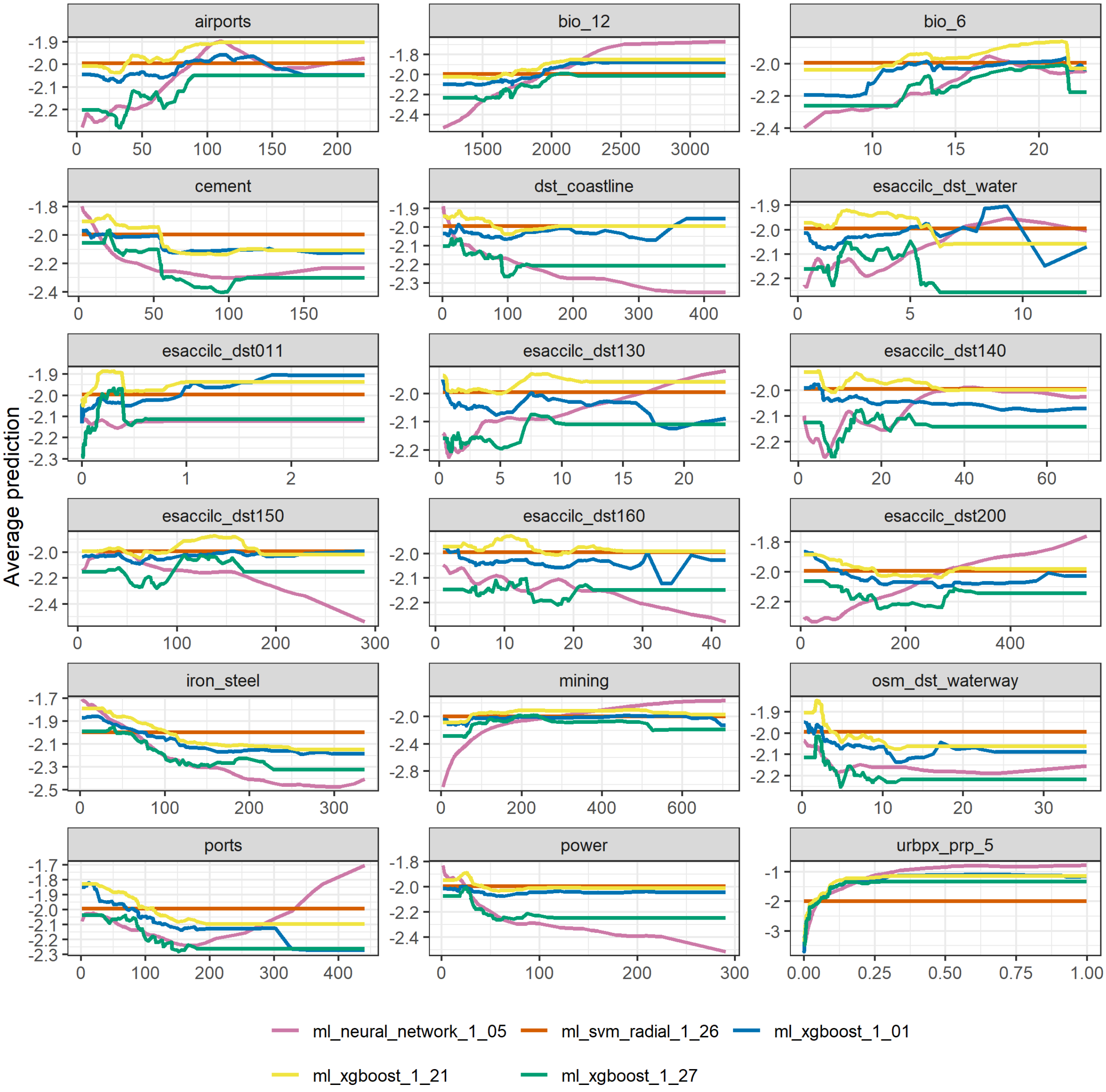

Fig. S18: Accumulated local effects plots for craft workers and operators. Results are presented for the five super learner model members with the highest weight.

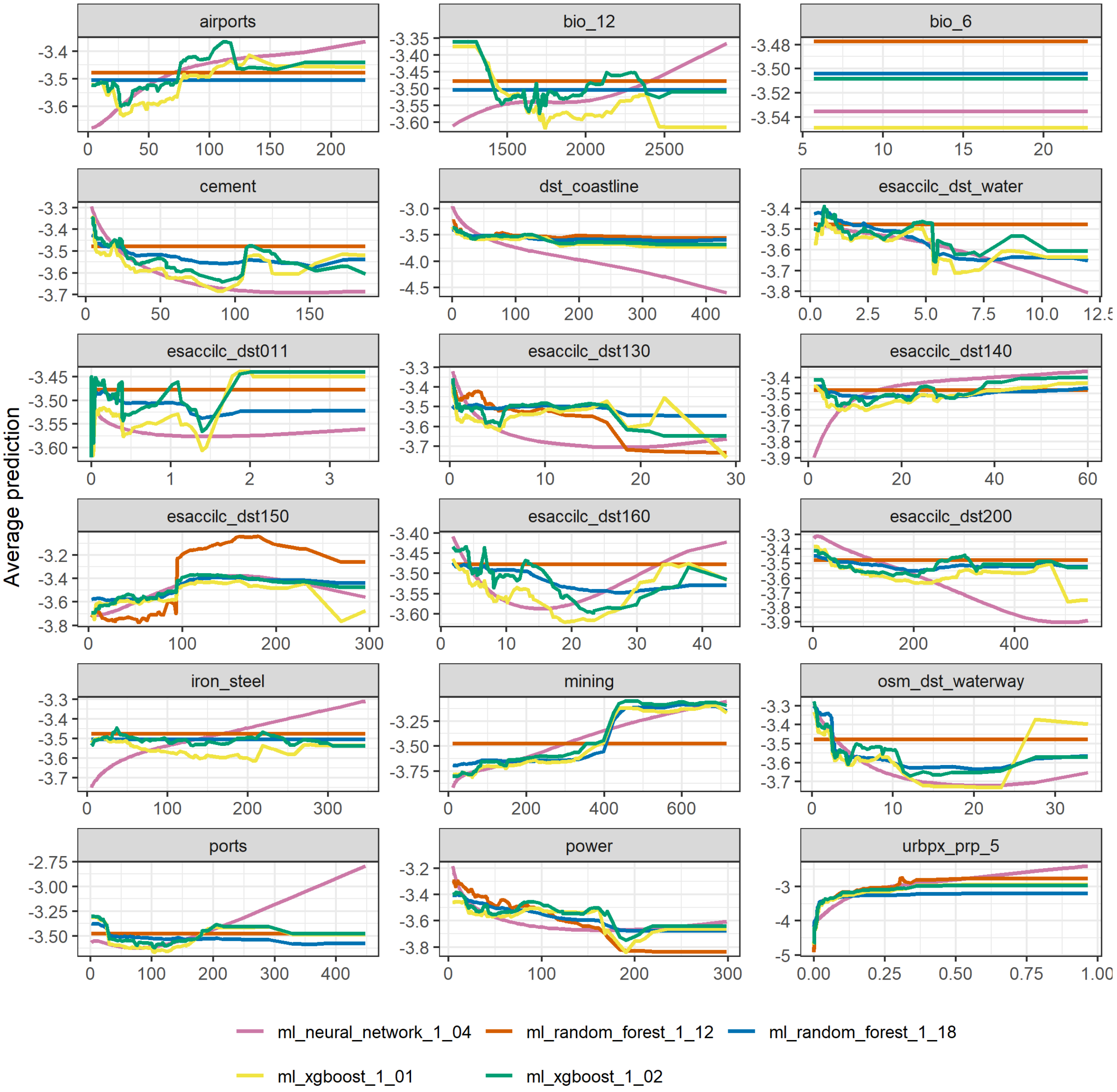

Fig. S19: Accumulated local effects plots for elementary occupations. Results are presented for the five super learner model members with the highest weight.

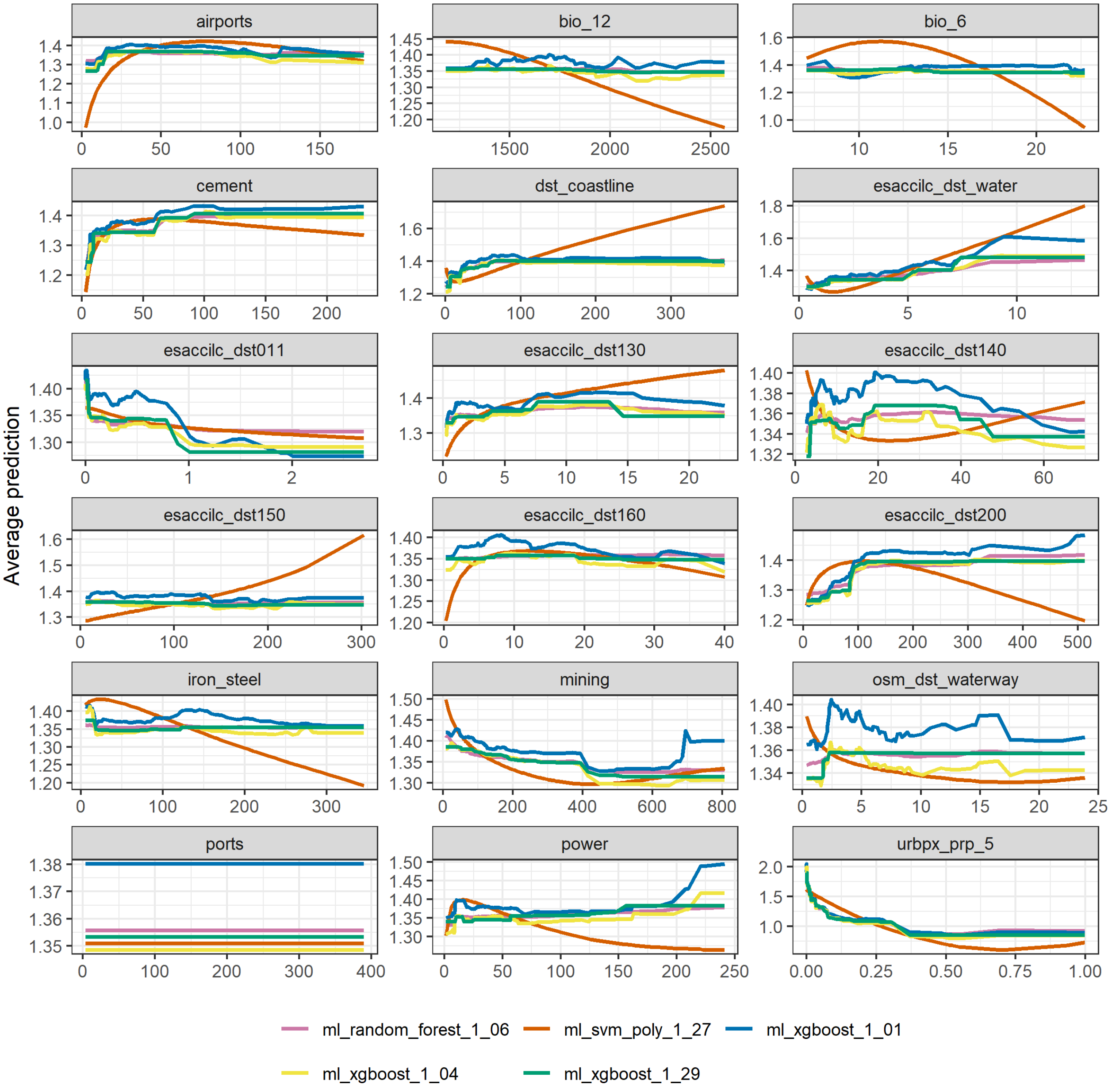

Fig. S20: Accumulated local effects plots for labour force participation rate. Results are presented for the five super learner model members with the highest weight.
